# The economic recession and civic participation: the curious case of Rotterdam's civil society, 2008–2013^1^

**DOI:** 10.1111/1468-4446.12691

**Published:** 2019-07-08

**Authors:** Gijs Custers, Godfried Engbersen, Erik Snel

**Affiliations:** ^1^ Erasmus University Rotterdam

**Keywords:** Civil society, neighbourhood research, volunteering, civic participation, economic recession

## Abstract

This paper investigates how the 2008–9 recession affected civic participation in disadvantaged and affluent neighbourhoods in the city of Rotterdam. We hypothesize that levels of civic participation may either diverge or converge across neighbourhoods with a different socioeconomic status. We build upon a recent wave of studies examining how civil society has been affected by the 2008–9 recession. Using five waves from the Rotterdam Neighbourhood Profile survey (*N* = 63,134; 71 neighbourhoods), we find converging trends in civic participation. Between 2008 and 2013, civic participation declined in affluent neighbourhoods but increased slightly in disadvantaged neighbourhoods. This convergence is partly due to the level of perceived problems in the neighbourhood and differences in the types of volunteering found in disadvantaged and affluent neighbourhoods. In addition, we argue that these converging trends can be better understood by considering the neighbourhood organizational infrastructure and local policy configurations. Next to examining the impact of the 2008–9 recession on civic participation, we contribute to research on civil society by comparing the UK and Dutch context.

## Introduction

How people and communities respond to economic hard times has long been of interest to sociologists (e.g. Bourdieu et al. 1999; Jahoda, Lazarsfeld and Zeisel 2017 [1971]; Putnam 2000; Wilson 1996). A recent wave of studies has examined how civil society was affected by the 2008–9 recession (Civil Exchange 2015; Clifford 2017; Jones, Meegan, Kennett and Croft 2016; Lim and Laurence 2015; Lim and Sander 2013; Rotolo, Wilson and Dietz 2015). An innovative study by Lim and Laurence (2015) shows that volunteering declined in the UK during the recession period and that this decline was steeper in disadvantaged communities. They suggest this varying effect of the recession across communities was a result of changes in organizational infrastructure and cultural norms. Their findings raise an important issue: do economic recessions unevenly affect civic involvement in different communities or areas and what mechanisms explain these differences?

Many scholars anticipated that the recession would have an uneven impact on civil society (Kisby 2010; Lindsey 2013; Lowndes and Pratchett 2012; North 2011; Uitermark 2015). They argue that, in times of recession, with the corresponding austerity policies, affluent communities with strong social capital are better equipped to respond to changes in the civil domain than disadvantaged communities with less social capital. A possible consequence is that civic participation declines more in disadvantaged communities than in affluent communities. However, when comparing their UK findings to the US context, Lim and Laurence (2015) emphasize the importance of national institutions and cultural factors in understanding differences in volunteering behaviour, implying the 2008–9 recession did not necessarily cause a divergence in volunteering or other forms of civic participation among more and less disadvantaged groups, neighbourhoods, or regions.

In this study we examine the impact of the economic recession in more detail by focusing on neighbourhoods. Specifically we look at trends in civic participation across 71 neighbourhoods in the Dutch city of Rotterdam between 2008 and 2013 (*N* = 63,134; 5 waves). As far as we are aware, this is the first time‐series analysis of rates of civic participation at the neighbourhood level.

A comparison between the UK and Dutch context is particularly interesting, because both countries faced austerity during the 2008–9 recession and a similar discourse on civil society and the welfare state emerged. In the UK politicians referred to the ‘Big Society’ as a way of encouraging participation whereas the Dutch version is called ‘*participatiesamenleving* (participation society)’. They are very similar in the sense that they combine goals of promoting ‘citizen involvement’, ‘localism’ and ‘responsibility’ with a retrenchment of the state in the public domain (Kisby 2010; Uitermark 2015). While today the term ‘participation society’ is omnipresent in public discourse on civil society in the Netherlands, the idea of Big Society has lost its traction in the UK. Yet, the underlying principles of both concepts remain present in public discourse and policy (Crisp 2015). In terms of research these similarities in austerity and discourse between the UK and the Netherlands reaffirm the need for empirical investigation into developments in civic behaviour, since similar conditions do not always result in similar behaviour.

Our research confirms this notion. In contrast to Lim and Laurence (2015), we find that rates of volunteering and neighbourhood involvement in different neighbourhoods in Rotterdam generally converged between 2008 and 2013. In affluent neighbourhoods civic participation declined (especially volunteering), in neighbourhoods with middle socioeconomic statuses (SES) civic participation remained more or less the same, and disadvantaged neighbourhoods saw a small increase in civic participation. In this light we can reformulate the issue we noted before: why does inequality in civic participation between neighbourhoods increase or decrease during an economic recession? In this paper we suggest several mechanisms that could explain variable trends in civic participation during an economic recession. These mechanisms include the need for local involvement in disadvantaged neighbourhoods, the uneven impact of austerity on civic organisations and local social policies.

This study makes multiple contributions to the literature on civic participation. In addition to investigating the impact of the 2008–9 recession on civic participation and comparing the UK and Dutch context, it also pays attention to the role of neighbourhood and policy factors. The analysis has a multilevel framework, since individual, neighbourhood, and time‐related variables must be taken into account. Our central research question reads: How can trends in civic participation across neighbourhoods with a different SES in Rotterdam between 2008 and 2013 be explained?

## Theoretical framework

Civic participation is a broad term referring to people's involvement in voluntary organizations and grassroots initiatives (Putnam 2000). Civil society is a sphere that is separate from the family, state and market, one in which people take collective action around shared interests, purposes and values (Corry 2010). In practice, multiple links exist between civil society and other spheres, something that is also theorized in this study. We are interested in two forms of civic participation, namely volunteering and neighbourhood involvement. We regard both volunteering and neighbourhood involvement as forms of collective action within the civil sphere.

Volunteering is frequently considered as an indicator of how ‘healthy’ civil society is. It refers to mutual aid, as when a group of people work together to achieve a common goal (Musick and Wilson 2008: 11). Volunteering shows whether people display altruistic behaviour in general.

Neighbourhood involvement is conceptualized as being active for the neighbourhood in any organized form. This definition includes a range of activities, such as participating in a neighbourhood association or organizing an event with a group of residents. Neighbourhood involvement differs here from the idea of ‘neighbouring’ in general (cf. Wilson and Son 2018), since it focuses more on formal and organized activities. In discussions about the ‘participation society’ the need for residents to engage with their local environment, both socially and physically, is consistently emphasized, underlining the importance of investigating neighbourhood involvement.

Volunteering and neighbourhood involvement are distinct but similar forms of civic behaviour since both are predominantly local and people engage in both of them for similar reasons (Dekker and de Hart 2009; Musick and Wilson 2008). Our theoretical explanations of civic behaviour can therefore be applied to both forms.

The theoretical framework is outlined as follows. First, we present a general theory of civic participation that helps explain trends in civic participation during the 2008–9 recession. Second, we describe characteristics of the recession and argue why, in combination with theory about civic participation, inequality in civic participation could increase during the recession. We then develop an opposite hypothesis, namely that inequality in civic participation will decrease during the recession, by providing more details on Rotterdam and its local policies.

### Individual employment and neighbourhood factors

Given that a recession causes widespread unemployment, we first review the influence of employment on civic participation. Mixed views exist about the relation between employment and civic participation (Lim and Sander 2013; Strauß 2008; Wilson 2000), since some studies suggest that work integrates people into social networks which foster civic participation (Musick and Wilson 2008; Rotolo and Wilson 2003), whereas other studies indicate that people with no or limited working hours (the unemployed, part‐time workers, retirees) devote more time to volunteering and similar activities (Dekker, Fouarge and Schils 2008; Markham and Bonjean 1996). In the Netherlands the latter view seems more valid, as people with more free time feel they have to ‘contribute to society’ and volunteering can provide access to the labour market (see Dekker and de Hart 2009).

Whether people are likely to participate in the civil domain is further influenced by the area in which they live. In the neighbourhood effects literature, several neighbourhood characteristics have been identified as explanations for differences in civic participation between neighbourhoods; differences that cannot be attributed to the individual characteristics of residents (van Ham et al. 2012). Although many studies have focused on the role of ethnic diversity (see van der Meer and Tolsma 2014), neighbourhood SES seems to be a more important contextual characteristic for explaining differences in social capital and civic behaviour (Bécares, Stafford, Laurence and Nazroo, 2011; Laurence 2009; Letki 2008; Tolsma, van der Meer and Gesthuizen 2009). Neighbourhood SES has particular relevance for our theoretical framework, since we hypothesize that levels of civic participation will diverge or converge according to the available socioeconomic resources in neighbourhoods (cf. Snel, Custers and Engbersen 2018).

Several scholars demonstrated that level of neighbourhood SES and the organizational infrastructure associated with it are key to explaining differences in levels of civic participation (Sampson 2012; Sampson, McAdam, MacIndoe and Weffer‐Elizondo 2005; Small 2009; Wilson 1987, 1996).^2^ The resources available in higher SES neighbourhoods – particularly the type of resources possessed by educated, middle‐class residents – have positive effects on participation because (a) residents can invest these resources (such as financial capital and knowledge) in the organizational infrastructure, for example churches, neighbourhood centres, neighbourhood watches and other associations (Clifford 2018), which in turn stimulates the participation of other residents; and (b) higher educated neighbours potentially have positive peer influences (Stoll 2001; see also Galster 2012 on neighbourhood mechanisms). Organizations play a pivotal role because they enable participation; the formal character of civic participation is derived from its institutionalized form (Musick and Wilson 2008). In this regard empirical studies show a positive relationship between SES and organizational involvement on the communal level (Sampson and Graif 2009; Sampson and Groves 1989).

Other studies provide a different perspective on the relation between neighbourhood SES and civic participation. A low level of neighbourhood SES can also spur civic participation, since the need for participation will be more urgent in low SES neighbourhoods (Gilster 2014; Perkins et al. 1990; Snel, Custers and Engbersen 2018; Swaroop and Morenoff 2006). Poor neighbourhoods are associated with problems such as litter, feelings of unsafety, crime, and deterioration. Such problems can trigger social action by residents, leading to more participation in neighbourhood activities.

An example of this needs‐perspective in Rotterdam is *Opzoomeren*. This community‐development policy originated in the late 1980s in a street named ‘Opzoomerstraat’ when residents became discontented with its deteriorated state and worked to improve the environment with the assistance of municipal funds (Uitermark 2015). Nowadays about 1,700 street groups across Rotterdam apply for Opzoomer funds, their goals being not only the improvement of the physical environment but also community‐orientated social events and language lessons (Opzoomer Mee 2018). Moreover, social professionals frequently provide assistance during Opzoomeren, meaning the state not only provides funds but is also actively participating (cf. de Graaf, van Hulst and Michels, 2015). This example illustrates that in the Netherlands – as opposed to the US context – the organizational infrastructure is partly maintained by the welfare state, thereby enabling equal opportunities for participation across neighbourhoods with a different SES (cf. Wacquant 2008).

### Possible negative effects of the economic recession

The neighbourhood perspectives provide preliminary insights into how organizations, and civil society more general, might have responded to the 2008–9 recession. After all, the recession has challenged the economic base of many organizations (Clifford 2017, 2018; Jones et al. 2016) and also the demand for volunteers (Lim and Laurence 2015; Rotolo, Wilson and Dietz 2015). The possible effects on civil society will become clearer after we discuss economic and public policy aspects of the recession.

In economic terms the recession led to high unemployment and austerity measures. Unemployment in Rotterdam rose from 5.8 per cent in 2008 to 12.6 per cent in 2014 (Table 1). The municipality initiated an austerity program in which roughly €150 million of policy budgets were cut for the period 2012–15 (Rotterdam Court of Audit 2011). This austerity program mainly targeted the departments of social welfare and care, which had an annual budget of approximately €420 million.

**Table 1:** Unemployment rate in Rotterdam, 2007–2016

	2007	2008	2009	2010	2011	2012	2013	2014	2015	2016
Unemployment rate %	6.6	5.8	6.7	8.0	8.2	10.5	12.3	12.6	12	11.3

*Source*: Dutch Statistics.

The recession is further associated with certain policy paradigms becoming more salient in public debate. The policy concepts Big Society and participation society are both characterized by a discursive emphasis on ‘responsibilization’ and ‘localism’ or ‘decentralization’ (Lowndes and Pratchett 2012; North 2011; Schinkel and van Houdt 2010). Responsibilization means citizens are primarily held responsible for personal and communal issues instead of the state. Localism or decentralization on the other hand signify that citizens and local communities should have more power and capability in organizing their public services, which have traditionally been provided by the nation state. In other words, responsibility for public services is transferred from the state to local government, communities and citizens.^3^

One possible consequence is that the amalgamation of austerity and discussions on policy led to a general decrease in civic participation during the 2008–9 recession. During an economic downturn civic organizations have more difficulties obtaining the amount of resources they need, since people tend to donate less money and public funds are cut. In turn, their opportunities to facilitate civic participation diminish. In addition, widespread and prolonged unemployment might lower people's sense of collective efficacy (Lim and Sander 2013: 16). Combined with political calls for ‘taking responsibility’, this may lead to widespread cynicism and thus dampen civic spirit and participation.

In line with the findings by Lim and Laurence (2015), many scholars have suggested that the likelihood of such detrimental effects on civil society might differ between communities (Civil Exchange 2015; Crisp 2015; Jones et al. 2016; Kisby 2010; North 2011; Uitermark 2015). Research from the UK shows that organizations experiencing the largest cutback in government resources were mainly located in deprived areas, where they serviced various disadvantaged groups (Civil Exchange 2015; Clifford 2017; Clifford, Geyne‐Rahme and Mohan 2013; Jones et al. 2016). Given that local communities bear increased responsibility for continuing their civic organizations, these findings strengthen the expectation that organizations in affluent communities with strong social networks are more capable of dealing with the challenges of the 2008–9 recession, whereas organizations in deprived communities with weak social networks were less capable of handling the cutback in resources (cf. Lindsey 2013).

These discrepancies disproportionally affect levels of citizen participation, because organizations form the base of participation; they provide the opportunities for people to volunteer or to become involved in neighbourhood issues (Sampson 2012; Small 2009). Lim and Laurence (2015) show that the probability of volunteering in disadvantaged communities decreased more than in affluent communities after the onset of the 2008–9 economic recession. This effect occurred at the communal level, meaning differences in volunteering could not be explained by people becoming unemployed or facing economic hardship on the individual level. They argue that this divergent effect is probably a result of changes in the organizational infrastructure, and not mere differences in individuals’ characteristics. Following this line of reasoning, it can thus be hypothesized that *the 2008–9 recession will have a stronger negative effect on civic participation when neighbourhood SES is lower (divergence hypothesis)*.

### Potentially equalizing effects

The UK studies show that the 2008–9 recession had a severe impact on civil society. However, an alternative theory predicts that civic participation would increase during an economic recession. In economic hard times people's needs are more difficult to meet through market or state mechanisms due to widespread unemployment or cutbacks in government services. More is expected from civic organizations who can mobilize volunteers and help those in need. Moreover, the recognition that people are struggling can heighten the sense of community and promote altruistic behaviour. The increased demand for help might thus lead to higher levels of civic participation in general (Lim and Laurence 2015; Lim and Sander 2013; Rotolo, Wilson and Dietz 2015).

Building on this premise, we can further expect that during an economic recession levels of civic participation will converge between disadvantaged and affluent neighbourhoods. The needs‐perspective we explicated before provides support for this hypothesis, since in disadvantaged neighbourhoods the need for participation is generally more urgent than in affluent neighbourhoods. A second argument relates to two Rotterdam policies, including the organizational infrastructure relating to civic participation and the Reciprocity Policy, which we will discuss in turn.

Rotterdam is the second most populous city in the Netherlands (over 600,000 inhabitants) and is inter alia known for its large socioeconomic inequalities, at least by Dutch standards (Hochstenbach 2017). It has a city‐wide organizational infrastructure, meaning there is a more or less equal distribution of civic organizations across the city (Uitermark 2012, 2015). According to Uitermark (2012), the municipality has from the 1980s onwards invested in umbrella organizations and professional support for vulnerable residents, immigrants, women, and other groups in all parts of the city, a governance figuration he refers to as ‘civil corporatism’. This figuration fits into a Dutch tradition of state involvement in the civil domain that highly values equal representation (cf. Salamon 1987), whereby civic initiatives and organizations aim to foster the participation of vulnerable residents (e.g. the unemployed or people with disabilities), in particular in disadvantaged neighbourhoods (de Graaf, van Hulst and Michels, 2015).

The city‐wide organizational infrastructure was not unaffected by the 2008–9 recession. Multiple public provisions such as neighbourhood centres and public libraries were closed and funds for civic associations and activities reduced (Bronsveld 2016; van der Zwaard and Specht 2013). Yet, despite the recession the municipality still offers various funding possibilities for civic groups, for example through Opzoomeren or other resources that are allocated across low, mixed and high SES neighbourhoods alike (Bronsveld 2016; Opzoomer Mee 2018). In line with the Dutch tradition of state involvement, the municipality's policy view is that neighbourhood organizations should be primarily run by local residents but that social professionals will help in those districts where residents are not sufficiently capable of managing themselves (Municipality of Rotterdam 2015). This policy view implicates that in districts with a less well‐developed civic base the local state maintains an organizational infrastructure that enables participation (Kullberg et al. 2015; cf. Wacquant 2008). Hence, neighbourhoods with a lower SES probably received more government support during the 2008–9 recession. This would imply that levels of civic participation in disadvantaged neighbourhoods were less negatively affected by the 2008–9 recession. Unfortunately, we are unable to incorporate the role of the organizational infrastructure in our analyses. Nevertheless, following Sampson (2011, 2012) we believe the presence of civic organizations has great theoretical relevance (see also discussion section).

The second policy to affect civic participation is known as the ‘Rotterdam Reciprocity Policy’. It requires social assistance recipients with a so‐called ‘large distance to the labour market’ – a Dutch expression to indicate persons who have little chance of obtaining formal employment – to do ‘something in return’ for the city, which frequently translates into performing ‘mandatory’ voluntary work (Bus, de Vries and van Zeele 2017).^4^ The Reciprocity Policy was gradually implemented during the period covered by our study: in 2011 an act of reciprocity was made mandatory in seven neighbourhoods, targeting about 12 per cent of all recipients and in 2013 the policy covered 14 neighbourhoods including about 21 per cent of all recipients (Municipality of Rotterdam 2014). Although the Reciprocity Policy covers all neighbourhoods in Rotterdam in 2018, during its introduction in 2011–13 the policy was targeted at low SES neighbourhoods that included large shares of the social assistance recipients in the city. During the economic recession civic participation by residents in low SES neighbourhoods may have increased at a higher rate compared to residents in higher SES neighbourhoods as a result of the Reciprocity Policy.^5^

Summing up, based on explanations relating to the needs‐perspective, the Rotterdam organizational infrastructure, and the Reciprocity Policy, we hypothesize that *the 2008–9 recession will have a stronger positive effect on civic participation when neighbourhood SES is lower (convergence hypothesis)*.

## Analytical strategy

The goal of our analysis is to test which hypothesis is most plausible, that is, whether civic participation diverged or converged across neighbourhoods with a different SES during the 2008–9 recession. In the next section we introduce the various data sources we used for our analyses and describe how our individual and neighbourhood factors are operationalized. Thereafter, we present a graph that shows the general trends in volunteering and neighbourhood involvement in Rotterdam between 2008 and 2013. We then test our interaction hypotheses by estimating multilevel regression models including individual, neighbourhood and time‐related variables. Our last step is to explore which factors explain our findings. We show how experiencing neighbourhood problems, associated with the needs‐perspective, is related to changing levels of civic participation. The role of the Reciprocity Policy is also considered. In addition, we indicate how different kinds of volunteering are related to changes in civic participation. Our analytical choices are further clarified in the results section.

## Data

We use five waves (2008, 2009, 2010, 2011 and 2013) from the Rotterdam Neighbourhood Profile survey, which covers 71 administrative neighbourhoods per wave.^6^ Unfortunately, no pre‐recession data are available, an issue we address in the discussion section. The cross‐sectional survey includes between 11,000 and 15,500 respondents depending on the wave. The Rotterdam Neighbourhood Profile serves as an instrument to monitor the ‘social and physical state’ of the city and includes questions about neighbourhood issues, social participation, health and labour market status (Municipality of Rotterdam 2018).

The respondents were selected by a stratified random sampling method in which neighbourhoods were the grouping level (samples were drawn from the population register). In addition, ethnic minorities such as Turks and Moroccans were oversampled to obtain representative response rates. The net response rates varied between 21 per cent and 23 per cent. The initial aggregated dataset included 65,486 respondents; after a listwise deletion of missing values (3.6 per cent of the sample), 63,134 respondents remain for analyses. For some categorical variables (i.e. education and employment status) an extra dummy was added for missing values instead of applying listwise deletion.

### Measurements

*Volunteering* is measured by asking respondents whether they were active (unpaid) for one or more organizations as a volunteer. A note elucidated that ‘unpaid’ means they can receive a reimbursement, but not a wage. Response categories were either yes (1) or no (0). *Neighbourhood involvement* is measured by the following question: have you been actively engaged in your own neighbourhood in the past 12 months, and if yes, in what way? Respondents could indicate whether they had volunteered (1), had contributed to the liveability of the neighbourhood (2), had been involved in local politics, policy or governance (3), and/or had contributed in any other way (4). For each response category examples of organized activities were mentioned. Responses were coded into being active for the neighbourhood (1) or not (0).

Our time variable that covers the recession period is a continuous variable (*Recession period (2008–2013)*). The year 2008 was coded zero and for every year the variable increases by one, up to four for 2013. The variable *neighbourhood problems* measures to which extent respondents find that there are many problems in their neighbourhood. The response categories were a 5‐point Likert scale that was coded ‘totally disagree’ (0) up to ‘totally agree’ (4). In addition, we include multiple independent variables such as education and self‐rated health which, as demonstrated in previous research, explain variations in civic participation. Information about these variables can be found in Table 2.

**Table 2:** Descriptive statistics

Variables	Mean	St. Dev.	Minimum	Maximum
Volunteering	0.229		0	1
Neighbourhood involvement	0.234		0	1
Education (ref. = low)	0.135		0	1
Middle low	0.242		0	1
Middle	0.267		0	1
High	0.326		0	1
Missing	0.029		0	1
Employment status (ref. = works > 12 h)	0.515		0	1
Economically inactive	0.380		0	1
Unemployed	0.091		0	1
Missing	0.014		0	1
Age	48.4	18.0	15	103
Age squared	2666.5	1843.1	225	10609
Gender (ref. = female)	0.428		0	1
Household status (ref. = single household)	0.358		0	1
Couple with no children	0.287		0	1
Couple with children	0.254		0	1
Single parent HH	0.083		0	1
Other	0.019		0	1
Ethnicity (ref. = autochthonous)	0.585		0	1
Turkish	0.067		0	1
Moroccan	0.042		0	1
Antillean	0.030		0	1
Surinamese	0.087		0	1
Cape Verdean	0.025		0	1
Other	0.164		0	1
Homeowner (ref. = renter)	0.561		0	1
Self‐rated health	2.284	1.060	0	4
Dutch proficiency	1.845	0.429	0	2
Religious attendance	0.800	1.416	0	4
Neighbourhood problems^a^	1.420	1.031	0	4
Neighbourhood SES	0	1	−2.385	2.031
Ethnic diversity	0.635	0.174	0.166	0.859
Residential turnover	0.105	0.039	0.041	0.354
*N* individuals	63,134			
*N* neighbourhood‐years	351			

Notes:

The variables age and age squared were recoded for the regression analyses so that the multilevel models converged. Age was divided by 10 and age squared by 1000.

a *N* = 58,459.

*Neighbourhood SES* is a scale constructed from four indicators measured at the neighbourhood level: the percentage of people with low income; the percentage receiving social assistance benefits; the unemployment rate; and the average level of disposable income. These data were provided by Research and Business Intelligence (OBI), the research department of the Rotterdam municipality, and are derived from Statistics Netherlands, Work and Income Rotterdam, and the Social Security Agency for Employee Insurance (UWV). A factor analysis with these four indicators indicated that one scale can be formed (loading scores > 0.84; Cronbach's alpha = 0.94), which was calculated based on standardized regression scores.

On the neighbourhood level we further control for the influences of ethnic diversity (see Savelkoul, Gesthuizen and Scheepers 2015) and residential (in)stability (Kasarda and Janowitz 1974). A Herfindahl Hirschman Index (HHI) measures the degree of *ethnic diversity* per neighbourhood. This index was calculated using data from the Municipal Personal Records Database, provided by OBI, which includes each share of nine ethnic groups per neighbourhood.^7^ Residential stability is measured by the degree of *instability*, which is the sum of all moves within, to and out of a neighbourhood divided by the total number of residents (see Municipality of Rotterdam 2018). This measure is like the HHI based on records from the Municipal Personal Records Database. For all neighbourhood variables the contextual data were taken from the same year as the year of the Neighbourhood Profile survey.

## Results

Our data cover the period between 2008 and 2013, which more or less captures the start of the 2008–9 recession and a large part of the economic downturn. Table 1 shows that the unemployment rate in Rotterdam gradually increased after 2008 and only slowly declined after its peak in 2014. These numbers indicate that the negative consequences of the recession increasingly manifested themselves during our period of study (cf. Lim and Laurence 2015).

**Figure 1:**
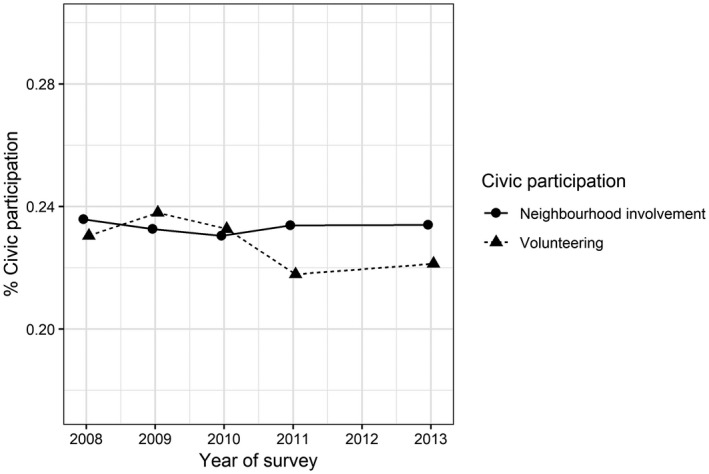
Trends in civic participation, 2008–2013

Looking at the general developments in civic participation during the 2008–9 recession, we observe no substantial changes (Figure 1). Neighbourhood involvement remained stable between 2008 and 2013, whereas volunteering declined slightly between 2009 and 2013 (by 1.7 per cent). Even though other studies have also reported stable rates of volunteering and other forms of civic engagement over a longer period (van Houwelingen and Dekker 2017; Rochester 2018), they typically do not consider that while there was an overall lack of change, some groups might have increased their participation while others participated less.

### Results from regression analyses

We next examine whether civic participation varies across neighbourhoods and time. Our dataset has a nested structure, since ‘neighbourhood’ and ‘time’ (year of survey) are both contextual levels. To obtain accurate standard errors, we estimate random slope models that account for this complex nesting structure.^8^ Following Schmidt‐Catran and Fairbrother (2016) we apply three‐level models that include years, neighbourhoods, and neighbourhood‐years as contextual levels.^9^

To test our interaction hypotheses, we follow a similar strategy as Lim and Laurence (2015). They included spline variables in their models, which are essentially linear time variables used to estimate whether trends in civic participation can be attributed to the 2008–9 recession itself and not to other factors such as random sampling variability or changes in demographic composition of neighbourhoods. Since we have no pre‐recession data, we include just one time variable covering the recession period (2008–2013).^10^ This time effect should vary between neighbourhoods with a different SES. Therefore, we estimate random slope models in which the slope of the time variable (i.e. recession period) is set random across neighbourhood SES.^11^ By studying the interaction between the time variable and neighbourhood SES, we are able to test whether there was a decline or rise in civic participation across disadvantaged and affluent neighbourhoods. We present the full models for volunteering and neighbourhood involvement, including the interaction term. All mentioned effects are statistically significant (*p *< 0.001) unless indicated otherwise.

Models 1 and 3 show the effects for a selection of variables on volunteering and neighbourhood involvement (see Table 3). Many effects, of which some are omitted to save space, are very similar in both size and direction, which we believe confirms that volunteering and neighbourhood involvement are similar forms of civic participation. Furthermore, the Cramer's V correlation between the dependent variables is 0.368, indicating they are closely related but still distinct.

**Table 3:** The effects of individual, neighbourhood, and time variables on civic participation (odds ratios and 95% confidence intervals)

Variables	Volunteering		Neighbourhood involvement	
	Model 1	Model 2	Model 3	Model 4
Employment status (ref. = works > 12 h)				
Economically inactive	1.918*** (1.860, 1.977)	1.904*** (1.844, 1.965)	1.336*** (1.278, 1.394)	1.327*** (1.268, 1.387)
Unemployed	1.779*** (1.702, 1.856)	1.797*** (1.717, 1.877)	1.377*** (1.301, 1.452)	1.409*** (1.331, 1.487)
Ethnic diversity	0.619** (0.322, 0.916)	0.613** (0.306, 0.920)	0.629** (0.292, 0.967)	0.570** (0.217, 0.924)
Residential turnover	0.490 (−0.357, 1.337)	0.489 (−0.385, 1.362)	1.337 (0.399, 2.275)	1.336 (0.361, 2.311)
Neighbourhood SES	1.028 (0.968, 1.089)	1.031 (0.969, 1.093)	0.912** (0.846, 0.978)	0.927* (0.858, 0.996)
Recession period (2008–2013)	0.976* (0.954, 0.997)	0.978 (0.954, 1.001)	0.987 (0.962, 1.011)	0.985 (0.956, 1.013)
Neighbourhood SES * Recession period	0.975*** (0.960, 0.989)	0.975*** (0.960, 0.990)	0.986* (0.973, 1.000)	0.988 (0.974, 1.002)
Neighbourhood problems		1.032** (1.010, 1.053)		1.141*** (1.121, 1.162)
Constant	0.104***	0.100***	0.116***	0.101***
*N* individuals	63,134	58,459	63,134	58,459
Log likelihood	−30,960.20	−28,954.38	−32,109.20	−30,129.03

Notes:

Models include the individual variables education (4 dummy categories), age, age squared, gender, household status (5 dummy categories), ethnicity (7 dummy categories), homeowner, self‐rated health, and religious attendance. Results are available upon request.

* *p* < 0.05

** *p* < 0.01

*** *p* < 0.001.

Being unemployed has a positive effect on volunteering (OR = 1.779) and neighbourhood involvement (OR = 1.377). The odds for the unemployed to volunteer, controlled for other characteristics, are 1.8 times higher than the odds for those working 12 hours or more per week. These findings confirm that in the Netherlands unemployment is positively related to civic participation.

Our main variable of interest is the interaction term between the recession period and neighbourhood SES. For volunteering the interaction term is negative (OR = 0.975; Model 1), indicating that the time effect is more negative in neighbourhoods with a higher SES. We find a similar, but slightly smaller effect for neighbourhood involvement (OR = 0.986, *p* < 0.05; Model 3). The significance levels of these interaction terms indicate that at least some variance in civic participation can be attributed to the effect of the recession itself and how it differs across neighbourhoods. Yet, given the size of our dataset, statistical significance may not be that meaningful here (see Wasserstein, Schirm and Lazar 2019). Considering the size of the odds ratios we observe these are just below 1, as are the values within the confidence intervals. This indicates that very modest interaction effects are present. For example, the size of the main effect of recession period for volunteering is 0.976 (*p* < 0.05; Model 1), meaning that in an average SES neighbourhood for every year the odds to volunteer are 0.976 times higher than the odds of the year before. Moreover, for every unit increase in neighbourhood SES (i.e. one standard deviation, see Table 2), the effect of recession period multiplies by 0.975 (cf. Buis 2010). Thus, in especially higher SES neighbourhoods the recession effect is more negative.

The magnitudes of the changes in civic participation across neighbourhoods are better understood when we depict the predicted probabilities, summarized for neighbourhood SES quintiles. For the lowest quintile the probability of volunteering increased by 2.6 between 2008 and 2013 (Figure IIa). For the second and third quintiles the probabilities remained stable, whereas the probabilities decreased for the highest two quintiles. In particular, neighbourhoods in the highest 20 per cent of the socioeconomic strata (fifth quintile) show a large decline: the probability of volunteering decreased by 5.2 per cent between 2008 and 2013. Figure IIb further illustrates that the probabilities of neighbourhood involvement also converged over time, albeit to a lesser extent than volunteering. The probability for neighbourhoods in the lowest quintile increased by 2.4 per cent, whereas the probability decreased by 2.4 per cent for the highest quintile.

Our key findings so far are the converging trends in civic participation between lower and higher SES neighbourhoods during the recession period. Although these changes are not dramatic, they are quite substantial given our relatively brief period of study. The changes in volunteering are larger than in neighbourhood involvement. The decline in volunteering in high SES neighbourhoods is especially noteworthy.

Based on Models 1 and 3 we assess that at least some of the observed changes can be attributed to the recession. We therefore conclude that the convergence hypothesis is more likely to be true than the divergence hypothesis. In the next sections we investigate how these findings can be explained given our theory and data.

### Changes in low SES neighbourhoods

The small increase in civic participation in low SES neighbourhoods requires more scrutiny, especially because it was logical to assume, based on several UK studies, that civic participation would decline in more disadvantaged areas. In our theoretical section we explained why deterioration in disadvantaged neighbourhoods would trigger civic action. When we add the variable ‘neighbourhood problems’ to our models (Table 3), we see that the more problems people perceive in their neighbourhood, the more likely they are to volunteer (OR = 1.032, *p* < 0.01; Model 2) or to be involved in the neighbourhood (OR = 1.131; Model 4). Moreover, Figure III shows that people in neighbourhoods with a lower SES perceive more problems on average. Neighbourhoods in the lowest three quintiles had an especially large increase in perceived neighbourhood problems since 2010. Together, these observations suggest that perceived problems in low and middle SES neighbourhoods partly explain why people became more civically active during the recession. This explanation seems particularly valid for neighbourhood involvement, because the odds ratio of neighbourhood problems is higher than for volunteering.

We also considered the Reciprocity Policy as a possible explanation for why civic participation could increase in low SES neighbourhoods. Since this policy's main goal is to increase volunteering among social assistance beneficiaries, we consider here whether volunteering rates rose among the unemployed.^12^ Table 4 shows a steady increase in the city's average rate of volunteering among the unemployed during the recession period. Among the unemployed in the lowest neighbourhood quintile the increase was small until 2011, but thereafter increased rapidly from 17.1 per cent in 2010 to 25.1 per cent in 2013. Remember that the Reciprocity Policy was implemented in 2011. Hence, it is likely to have affected volunteering in low SES neighbourhoods to some extent. At the same time, Figure IIa indicates that volunteering also changed before 2011. Clearly, the Reciprocity Policy is not the only mechanism that explains changes in volunteering in low SES neighbourhoods.

**Table 4:** Average levels of volunteering for the unemployed in Rotterdam, 2008–2013

	2008	2009	2010	2011	2013
City mean %	18.6	19.1	22.5	23.9	25.6
Lowest quintile %	15.3	16.4	17.1	21.0	25.1

### Changes in high SES neighbourhoods

We suspect that any decline in civic participation in high SES neighbourhoods could be the result of differences in types of civic participation, as Clifford (2017) for instance shows that the revenues of certain charity sectors (e.g. culture and recreation) were more affected by austerity policies than other charity sectors such as international development. In addition, people with different SES characteristics tend to engage in different types of associations and activities (van Ingen and van der Meer 2011; van der Meer, Grotenhuis and Scheepers 2009). Unfortunately, our data only contain information on what kind of volunteering respondents did in 2008, making it impossible to analyse changes in volunteer type during the recession. However, combined with our theoretical framework these figures may still provide insights into these changes.

Table 5 shows for which organizations people were active as a volunteer (multiple answers were possible). Some types of volunteering, such as those related to religion, hardly varied across neighbourhood SES, whereas neighbourhoods greatly differed on other types (cf. Clifford 2012). Volunteering for sports associations is mostly carried out in higher SES neighbourhoods (fourth and fifth quintile; 28.3 per cent and 28.6 per cent) while the lowest SES neighbourhoods (first quintile) distinguish themselves by the large proportion of volunteers in neighbourhood organizations (19.3 per cent). These differences in types of volunteering might explain the decline in higher SES neighbourhoods – and the converging trends in general – as follows. During an economic recession it might be more accepted to withdraw from civic life related to sports, since these associations serve leisure needs. People have other priorities, devoting their time to more pressing needs such as work or family care. On the other hand, neighbourhood organizations are more likely to serve local needs regarding liveability, which are probably more pressing during a recession (see also Figure III). Thus, this type of volunteering might continue during a recession due to a greater sense of urgency. In the case of Rotterdam such organizations were also more likely to be supported by the municipality than leisure organizations (Municipality of Rotterdam 2015), although hard evidence is lacking here.

**Table 5:** Types of volunteering for associations split by neighbourhood SES, 2008

Types of associations	Lowest quintile	2nd	3rd	4th	Highest quintile	Total
	%	%	%	%	%	%
Sports association	14.9	19.7	18.6	28.3	28.6	23.0
Religious association	20.3	19.7	21.9	19.1	18.3	19.7
School or pre‐school related	13.7	12.6	10.5	13.4	15.5	13.3
Organizations with societal goals	10.3	14.1	12.9	10.8	13.2	12.3
Neighbourhood centre or association	19.3	12.6	9.4	9.5	8.5	11.3
Elderly related	6.8	7.8	10.7	10.9	8.5	9.1
Music or theatre related	3.9	8.2	9.0	10.1	7.3	7.9
Hobby association	7.8	4.8	4.6	5.6	6.2	5.8
Youth related	4.6	5.2	4.2	4.3	3.8	4.4
Political organization	4.6	4.5	4.4	3.6	4.4	4.3
Union or professional related	2.7	4.3	4.4	4.3	5.0	4.3
*N* individuals	409	462	456	576	682	2,585

Note:

Multiple answers were possible.

## Conclusion

This study shows that civic participation across disadvantaged and affluent neighbourhoods in Rotterdam was more likely to converge than diverge during the 2008–9 recession, thereby providing different findings than previous studies on this topic (e.g. Lim and Laurence 2015). We started by hypothesizing why during the 2008–9 recession civic participation could either diverge or converge across neighbourhoods with a different SES. Based on a large dataset we observed small increases in volunteering and neighbourhood involvement in disadvantaged neighbourhoods between 2008 and 2013 and a decline in affluent neighbourhoods, especially for volunteering. In this section we summarize our explanations for these findings that have empirical ground.

We should first recognize that our models indicated that some variation in civic participation during the recession could be attributed to the effect of the recession itself and its variation across neighbourhood SES, but these effects were rather small. In other words, we should not overemphasize the magnitude of our findings. On that note, our empirical evidence offers several explanations.

Looking at why civic participation slightly increased in disadvantaged neighbourhoods, our analyses provide some support for the needs‐perspective (e.g. Swaroop and Morenoff 2006). Perceived problems in the neighbourhood were positively associated with civic participation, especially for neighbourhood involvement. During the recession period the amount of perceived problems increased in lower SES neighbourhoods, indicating that an increase in problems probably stimulated involvement in these neighbourhoods.

Another explanation for the small increase in volunteering in lower SES neighbourhoods is related to the Reciprocity Policy. This policy has been gradually implemented since 2011, starting in low SES neighbourhoods (Bus, de Vries and Zeele 2017). According to this policy, social assistance recipients are ‘obligated’ to perform voluntary work. Although the share of targeted people was relatively small, it probably had some effect on the observed trend in volunteering, partly because unemployed people had a higher probability of volunteering compared to employed people.

A second outcome is the decline in civic participation in affluent neighbourhoods, particularly volunteering. We argued this decline might be related to the types of volunteering. Residents in higher SES neighbourhoods volunteer more often than those living in low SES neighbourhoods for sports associations (almost 30 per cent). During a recession it is perhaps more acceptable to withdraw from this kind of volunteering because people have other non‐leisure priorities.

## Discussion

Next to the empirical explanations, we propose additional mechanisms that may explain the observed trends in civic participation. One mechanism is the organizational infrastructure of Rotterdam. The small increase in civic participation in disadvantaged neighbourhoods is somewhat counterintuitive, especially given the findings from the UK where disadvantaged areas seem to be most severely impacted by the 2008–9 recession (Civil Exchange 2015; Clifford 2017; Clifford, Geyne‐Rahme and Mohan 2013; Jones et al. 2016; Lindsey 2013). We proposed that the rate of participation in disadvantaged neighbourhoods is partly explained by the municipality's policy of supplying basic civic provisions in less advantaged neighbourhoods during times of austerity (Municipality of Rotterdam 2015; cf. Salamon 1987).

Another mechanism potentially explains why civic participation declined in affluent neighbourhoods. The argument here is that organizations in affluent neighbourhoods might experience more difficulties mobilizing resources and volunteers in times of hardship. They depend more on private contributions than organizations in disadvantaged neighbourhoods (Clifford 2012; Clifford, Geyne‐Rahme and Mohan 2013). Clifford (2018: 1585) shows that disadvantaged neighbourhoods have a higher rate of charity dissolution than affluent neighbourhoods, but this difference was narrowed during the 2007–2011 period. Contributors to organizations in affluent neighbourhoods might have reduced their donations during the economic recession, limiting the daily operations of these organizations and increasing their risk of dissolution. As a result, there would have been fewer opportunities for civic participation in affluent neighbourhoods.

We conclude by mentioning two limitations to the study. First, we could not take pre‐recession developments in civic participation into account. We cannot be certain that the observed trends are actually a result of the 2008–9 recession. Trends in civic participation could have gone up or down before. Other Dutch studies have reported quite stable rates of volunteering during economic booms and downturn (e.g. van Houwelingen and Dekker 2017), yet such studies have to our knowledge not investigated how underlying patterns of participation develop during economic recession – the general levels of civic participation were also stable in our study (Figure 1). Based on our theory, the empirical evidence, and the recession's severe impact, we are quite confident our results are related to the 2008–9 recession.

We were further limited in assessing the impact of factors like the neighbourhood organizational infrastructure (cf. Sampson et al. 2005) or austerity policies directly, because they are difficult to operationalize and data are scarce. Instead we focused on how the effect of ‘time’ varied across neighbourhoods with a different SES, whereby neighbourhood SES served as a proxy for the resources to which residents have access (cf. Sampson and Graif 2009). Ideally, we would have investigated directly how the structure of the organizational infrastructure (e.g. funding for neighbourhood organizations) affects levels of civic participation in different areas. Nonetheless, such intricacies demonstrate the importance of sound theory that can explain complex processes. Perhaps the most important lesson from our study is that empirical scrutiny is needed to determine whether similar conditions – referring to the recession and the policy concepts Big Society and participation society – produce similar outcomes.

**Figure 2:**
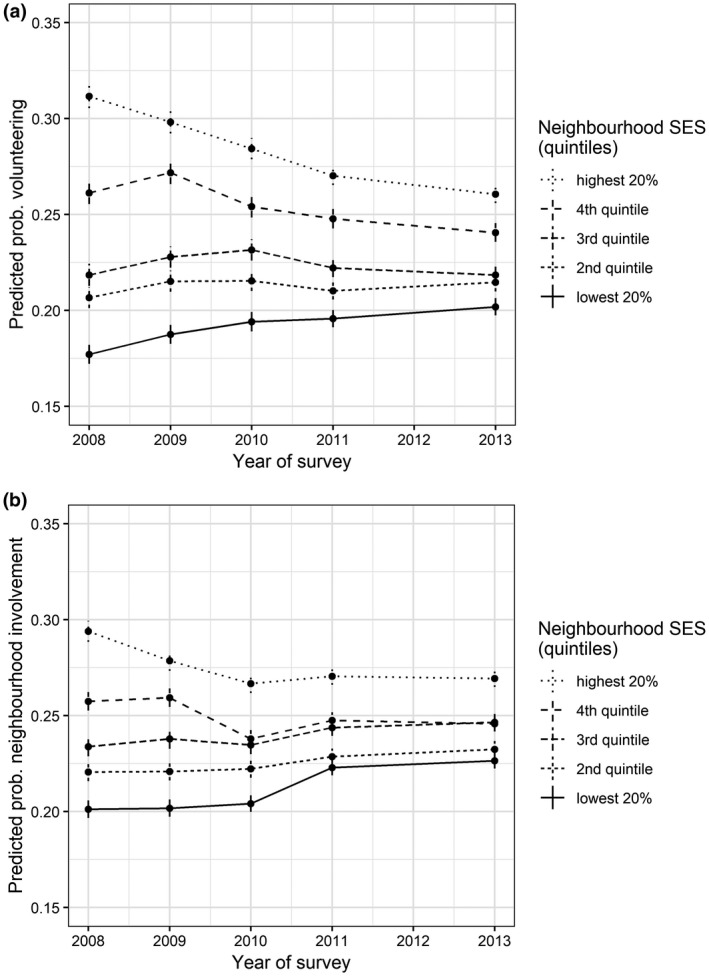
(a) Predicted probabilities for volunteering split by neighbourhood SES, 2008–2013. (b) Predicted probabilities for neighbourhood involvement split by neighbourhood SES, 2008–2013

**Figure 3:**
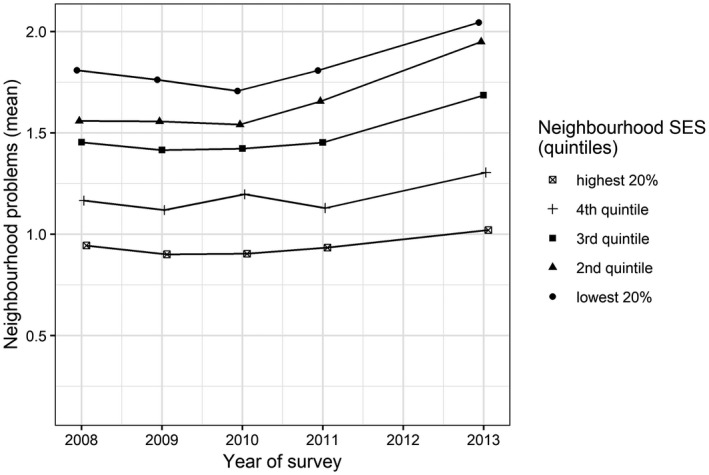
Trend in perceived neighbourhood problems (mean score) split by neighbourhood SES, 2008–2013

## Notes

2 An important difference is, however, that Sampson et al. (2005) mainly focused on collective civic events and not on individual participation.

3 The policy shifts and associated discourses are of course more complex than we are able to discuss here. In general it can be said that trends towards ‘responsibilization’ and ‘localism’ or ‘decentralization’ have been present for multiple decades (e.g. North 2011). In this regard the economic recession likely served as a catalyst for popularizing these policy paradigms.

4 Considering the participation society, this policy can be interpreted as making recipients more responsible for their own welfare (see also van Eijk 2010).

5 Due to the mandatory nature of the Reciprocity Policy the hypothesized increase cannot primarily be attributed to the intrinsic civic engagement of social assistance recipients. An evaluation of the Reciprocity Policy shows that about 75 per cent of the participants provide positive feedback on the scheme. The main reason for this positive feedback is that the scheme effectively counters social isolation (Bus, de Vries and van Zeele 2017).

6 Due to recent municipal expansion, the waves 2008 (69 neighbourhoods), 2009 (70 neighbourhoods) and 2010 (70 neighbourhoods) contain less than 71 neighbourhoods. An average neighbourhood has about 9,000 residents. The smallest neighbourhood contains 1,000 residents, whereas the largest neighbourhood contains 25,000 residents.

7 The nine ethnic groups are: autochthons, Turks, Moroccans, Surinamese, Cape Verdeans, Antilleans, other EU, other Western, and others.

8 For example, Lim and Laurence (2015) did not account for the multilevel structure of their data, which likely biased their estimates.

9 Model F in Table I on page 3 of their paper displays our model structure.

10 Spline variables are usually used to model a certain break in a longer trend (cf. Hout and Fischer 2002), something we are unable to do due to lack of pre‐recession data. We also estimated our models with ‘time’ as dummy variables instead of a linear variable and these models produced similar results.

11 The multilevel logit models were estimated in R, using the glmer function from the lme4 package (maximum likelihood fit).

12 The policy aims to increase institutional participation among the unemployed, i.e. participation in formal and mainly larger organizations. Therefore, we particularly focus on volunteering here and not neighbourhood involvement.
